# The Role of Th17 in Neuroimmune Disorders: A Target for CAM Therapy. Part III

**DOI:** 10.1093/ecam/nep064

**Published:** 2011-06-16

**Authors:** Aristo Vojdani, Jama Lambert, Gottfried Kellermann

**Affiliations:** ^1^Immunosciences Lab., Inc., Los Angeles, CA 90035, USA; ^2^NeuroScience, Inc., Osceola, WI 54020, USA

## Abstract

Abundant research has mapped the inflammatory pathways leading to autoimmunity and neuroinflammatory disorders. The latest T helper to be identified, Th17, through its proinflammatory cytokine IL-17, plays a pathogenic role in many inflammatory conditions. Today, healthcare providers have a wealth of anti-inflammatory agents from which to choose. On one hand, pharmaceutical companies market brand-name drugs direct to the public and physicians. Medical botanical knowledge, on the other hand, has been passed down from generation to generation. The demands for natural healing therapies have brought corresponding clinical and laboratory research studies to elucidate the medicinal properties of alternative practices. With a variety of options, it can be difficult to pinpoint the proper anti-inflammatory agent for each case presented. In this review, the authors highlight a vast array of anti-inflammatory medicaments ranging from drugs to vitamins and from botanicals to innate molecules. This compilation may serve as a guide for complimentary and alternative healthcare providers who need to target neuroinflammation driven by Th17 and its inflammatory cytokine IL-17. By understanding the mechanisms of anti-inflammatory agents, CAM practitioners can tailor therapeutic interventions to fit the needs of the patient, thereby providing faster relief from inflammatory complaints.

## 1. Introduction

Parts I and II of this series detail the destructive Th17 inflammatory pathway. Here, we present ways to intervene. The absence of Th17 cells can lead to a decrease in proinflammatory cytokines. Thus, to achieve the end goal of a balanced immune response, CAM practitioners may target components along the pathway leading from naïve T-cell differentiation to Th17, thereby lowering the levels of Th17 and IL-17. Treatment may include enhancing immunity by administering an agonist, or depressing Th17 cell function by introducing an antagonist of ROR*γ*t. The CAM protocol of ROR*γ*t agonist therapy may be applied to cancer patients when up-regulation of the immune response is needed. Intervention in autoimmune disorders or neurodegenerative diseases such as irritable bowel disorder, arthritis, diabetes and multiple sclerosis may include the use of antagonists to ROR*γ*t. Current methods, outlined here, include a choice of medicines ranging from pharmaceuticals to the integrative practice of intravenous therapy, and from natural plant extracts to innate molecules. Whether mainstream or alternative in practice, sometimes the most effective intervention includes a combination of these anti-inflammatory agents that is suitable for CAM.

## 2. Targeting Th17, ROR*γ*t Using Medications and Pharmaceuticals

General, or specific, medications have been or are being developed to reduce the symptoms of inflammatory diseases. Neuroprotective medications currently used for blocking microglial activation include minocycline or dextromethorphan [1]. The anti-inflammatory mechanisms of minocycline [2, 3] and dextromethorphan [4, 5] are unknown at this time; however, these products have been shown to be effective against neuroinflammation. An antibiotic that inhibits the response to IL-2 and thereby blocks the activation of both T cells and B cells, rapamycin, now called sirolimus, is used to combat autoimmunity and to prevent acute graft rejection [6]. To protect intestinal microflora and prevent further inflammatory damage, the use of antibiotics must be used in conjunction with a probiotic program.

Medications are not always favored; some practitioners prefer to incorporate intravenous therapies for their patients with chronic inflammatory disorders. Insulin-like growth factor-1 (IGF-1) is principally produced by the liver and circulates in the blood. Decreasing with age, a lack of IGF-1 is found in degenerative arthritis, septic shock, cardiovascular disease and inflammation of the bowel [7]. Treatment with intravenous immunoglobulin (IVIG), a plasma protein replacement therapy, uses immunoglobulins extracted from multiple healthy blood donors, is in use to decrease the severity of autoimmune disease [8, 9]. However, since IGF-1 treatment may increase growth hormone and growth factor, this should be the last choice [10].

The use of anti-cytokine treatments that target inflammation in autoimmune disorders is gaining popularity. Recombinant soluble TNF receptor, etanercept (Enbrel), binds to TNF and blocks inflammatory activity, while infliximab (Remicade) and adalimumab (Humira) are monoclonal antibodies specific for TNF, thus also binding to TNF and blocking inflammatory activity [11–14]. These anti-cytokine therapies are not specific and their long-term use can result in the development of immune complexes [15]. Pharmaceutical reviews by Drugs.com, an independent Internet source for consumers and healthcare providers, offer warnings associated with anti-cytokine medications that include drug interactions, suppressed immunity, exacerbation of heart disease and susceptibility to serious infections. These are just a few examples from a laundry list of potential problems. Full investigation into drug interactions, side effects and possible dire consequences of long-term use needs to be performed before the onset of pharmaceutical, anti-cytokine treatment.

## 3. Targeting Th17 through the ROR*γ*t Pathway

Possible targets for the suppression of Th17 production include four components: ROR*γ*t, TGF-*β*, IL-6 and NF-*κ*B. Inhibition of NF-*κ*B promotes the production of proinflammatory cytokines and can result in an improved balance between pro- and anti-inflammatory cytokines. Inhibiting IL-6 production, which is required for the induction of ROR*γ*t, may result in the inhibition of Th17 and the production of IL-17 cytokines. Enhancing the production of TGF-*β*, as this cytokine can influence transcription FoxP3, can differentiate the T cell into a regulatory T cell (T_reg_). Therefore, down-regulating ROR*γ*t, the transcription factor responsible for T-cell differentiation to Th17, will result in the reduction of the number of Th17 cells and therefore their cytokines. Overall, manipulation of the pathway toward the production of IL-17 can result in the inhibition of an inflammatory cascade.

### 3.1. Targeting ROR*γ*t

Retinoic acid-related orphan receptor (ROR)*γ*t is the key that unlocks the differentiation of naïve T-cells to effector Th17 cells. ROR*γ*t is expressed exclusively by immune system cells, double positive CD4^+^CD8^+^ thymocytes [16]. Interestingly, both IL-6 and TGF-*β* are individually capable of expressing small amounts of ROR*γ*t; however, neither can singly induce IL-17-secreting Th17. According to Batten et al. [17], the over-expression of this transcription factor induces production of Th17 and its effector cytokine IL-17; conversely, a deficiency of ROR*γ*t nearly halts T-cell differentiation to Th17. Therefore, by eliminating the key that unlocks IL-17-expressing Th17 cells, CAM practitioners may turn around the inflammatory response by targeting ROR*γ*t cells in their patients presenting with neuroimmune inflammatory disorders.

High levels of ROR*γ*t are present in inflammatory diseases, autoimmune disorders and food allergies. Treatment of immune-mediated diseases characterized by the presence of inflammatory cytokines, such as inflammatory bowel diseases, multiple sclerosis, arthritis, dry eye autoimmune disease and diabetes, may involve ROR*γ*t antagonists, while administration of agonists to ROR*γ*t may be helpful in the treatment of infectious diseases and boosting anti-tumor immunity [16, 18]. By the enhancement of mucosal immunity or the increase in the number of T cells reactive to a specific antigen, ROR*γ*t-developed Th17 can protect the host from infection.

The proximity of ROR*γ*t to Th17 in the development of IL-17 makes it a pivotal step in the immune response. When Th17 protection is necessary, the up-regulation of ROR*γ*t would be beneficial. On the other hand, if chronic inflammation exists, the production of IL-17 should be minimized, and thus the suppression of ROR*γ*t is desirable.

## 4. Targeting Th17, ROR*γ*t Using Complimentary and Alternative Agents

Due to risks associated with long-term use, undesirable side effects and the ineffectiveness of healing properties exhibited by many pharmaceutical agents, more and more patients suffering from chronic inflammatory disorders are turning to complementary and alternative medicine therapies. CAM practitioners have a wealth of natural alternatives to reduce inflammation. Previous authors of articles from *Evidence-Based Complimentary and Alternative Medicine* and other journals have provided an excellent insight into anti-inflammatories, such as Moutan Cortex (root bark of *Paeonia suffruticosa Andrews*) featured by Chun et al. [19] and later by Wu and Gu [20]; *Perillae fructus* (perilla seed) by Yim et al. [21], atractylenolide I (extracted from largehead *atractylodes rhizome*) by Liu et al. [22], and Chen et al. [23] examination of Tibetan medicated-bath therapy. Additional published natural anti-inflammatories that have been shown to inhibit the production of proinflammatory cytokines include *Opuntia humifusa* Raf. [24], total flavonoids of *Fructus Chorspondiatis* [25], *Artemisia annua* L. [26], *Kummerowia striata (Thunb.) Schindl* [27] and a mini review of plant extracts by Talhouk et al. [28] focusing on the anti-inflammatory properties of bioflavonoids (Table 1).

Indeed, the antioxidant activity and inflammatory response of flavonoids, or bioflavonoids, are of interest due to their therapeutic uses as anti-allergic, anti-cancer activity, anti-microbial and anti-inflammatory in the prevention or maintenance of various disorders. Natural antioxidants remove damaging free radicals and inhibit other oxidation reactions. By using different routes during inflammatory responses, flavonoids are able to block a range of known molecules, notably NF-*κ*B, inducible nitric oxide synthase (iNOS), cooxygenase (COX) and 5 lipoxygenase enzymes, resulting in the down-regulation of inflammatory cytokines such as IL-1*β*, IL-6 and TNF-*α*.

The list of published studies elucidating CAM therapeutic results against inflammatory responses is enormous. In the interest of space, we will confine our review to well-documented, natural substances that have been demonstrated to modulate Th17 or IL-17 as they are implicated in many neuroimmune, autoimmune and allergic disorders.

### 4.1. Targeting Th17, ROR*γ*t, Using Retinoic Acid, Vitamin D_3_, Resveratrol and Probiotics

Retinoic acid, the oxidized form of vitamin A, has long been viewed as important to both the innate and the acquired immune systems. The transcription factor ROR*γ*t, which if overexpressed, induces T-cell differentiation to Th17, belongs to a retinoid nuclear receptor superfamily [17]. Kim [29] provides an excellent review of published research data referencing the regulation of regulatory T cells by retinoids. Recently, researchers have recognized retinoic acid's regulation in immune responses [17, 30]. It was shown to suppress memory cell IFN-*γ* production and increase IL-4 secretion [17]. Mucida et al. [30] completed a study that measured the effect of retinoic acid on Th17 cell development both *in vitro* and *in vivo*. In each case, retinoic acid greatly reduced ROR*γ*t expression, which resulted in a measurable reduction of Th17 mucosal T cells. Further, all-trans retinoic acid (ATRA) has been reported to inhibit Th17 production and to promote FoxP3 expression, thereby affecting the Th17/T_reg_ polarization [17]. Retinoic acid's importance to the immune system cannot overshadow its promotion of glial and neuronal differentiation. Within the CNS (central nervous system), retinoic acid has been shown to enhance axonal regeneration of differentiated retinal ganglion cells and peripheral sensory neurons [31]. Therefore, supplementation with low doses of vitamin A may be useful for treatment of neuroimmune disorders.

### 4.2. 1*α*,25-Dihydroxyvitamin D_3_

The active metabolite of vitamin D_3_, 1*α*,25-dihydroxyvitamin D_3_ (1,25(OH)_2_D_3_), which can prevent the maturation of dendritic cells resulting in tolerogenic dendritic cells with increased potential to induce regulatory T cells is a proven potent inhibitor of NF-*κ*B expression. Taher et al. [32], using a mouse model of allergic asthma, demonstrated that the 1,25(OH)_2_D_3_ augmentation of immunotherapy resulted in increased serum levels of TGF-*β*, and thus prevented the Th2-driven development of asthma manifestations. In a study comprising patients with multiple sclerosis, researchers saw a rise in TGF-*β* levels following a 6-month supplementation phase of vitamin D [33]. Interestingly, there were no changes in TNF-*α*, IFN-*γ* and IL-13 levels. Vitamin D_3_ acts by binding in signal transduction pathways such as the regulation of intracellular concentrations, protein kinase C activity, phospholipids metabolism and cyclic nucleotide formation [33]. CAM practitioners, by specifically targeting an increase of TGF-*β*, should consider the effectiveness of vitamin D_3_, as it has been shown to reduce autoimmune incidences of cardiovascular disease [34], diabetes [35, 36], rheumatoid arthritis [37, 38] and lupus [39].

### 4.3. Resveratrol

A phytoalexin produced by several plants when under bacterial or fungal attack; resveratrol has become a popular supplement due to the French paradox. It has been suggested that the resveratrol found in the skin of red grapes explains the low incidence of cardiovascular disease in France where wine is consumed almost daily [40]. Indeed, immunostimulant resveratrol has been demonstrated to exhibit antioxidant properties and anti-inflammatory characteristics; it stimulates natural killer cell activity and exercises anti-tumor properties through the suppression of the NF-*κ*B signaling pathway [41]. Gonzales and Orlando [42] studied the effects of resveratrol and curcumin on the NF-*κ*B-mediated cytokine expression in adipocytes. They concluded that both supplements were able to inhibit TNF-*α*-activated NF-*κ*B signaling in adipocytes and thereby reduced TNF-*α*, IL-1*β* and IL-6 cytokine expressions. The authors of this study suggest the potential use of resveratrol and curcumin in the development of therapeutic protocols aimed at alleviating the low-level, chronic inflammation often seen in obese patients with a propensity toward cardiovascular disorders and insulin resistance associated with type 2 diabetes mellitus. In cases of neuroinflammation, the small molecules of resveratrol can easily pass through the blood-brain barrier where they are shown to be neuron-protective. Resveratrol stimulates the production of nicotinamide adenine dinucleotide (NAD), and therefore, is used to slow the progress of amyotrophic lateral sclerosis [43]. The neuroimmune protective characteristics of resveratrol make it an attractive and versatile option for CAM practitioners.

### 4.4. Non-Pathogenic Probiotic Bacteria

The human gut is a home to a vast consortium of symbiotic bacteria. Members of this complex microflora metabolize dietary substances, such as plant polysaccharides, that are otherwise indigestible by their human hosts. Interest in the gut flora has led to numerous investigations to demonstrate that there are beneficial and potentially harmful microorganisms in the intestine, and that the one could be used to influence the activities of the other. These findings led to the “probiotic” concept, originally used to describe microbial feed supplements that stimulate the growth of farm animals. Now, the use of live microbes as dietary supplements has been extended to humans [44]. The genera most commonly used in probiotic preparations are *Lactobacillus, Bifidobacterium, Streptococcus, Lactococcus* and some fungal strains.

Some of the health benefits which have been claimed for probiotics include the following: improvement of the normal microflora, prevention of infectious diseases and food allergies, reduction of serum cholesterol, anticarcinogenic activity, stabilization of the gut mucosal barrier, immune adjuvant properties, alleviation of intestinal bowel disease symptoms and improvement in the digestion of lactose in intolerant hosts. The beneficial effects of probiotic bacteria in relation to the augmentation of the immune system and cytokine production were summarized in a mini review by Galdeano et al. [44]. Pochard et al. [45] experimented with lactic acid bacteria and concluded that its administration enhanced the secretion of IL-12 and IFN-*γ*, thereby modulating the Th1/Th2 balance in cases of allergy. Ménard et al. [46] determined that metabolites secreted by lactic acid bacteria exerted an anti-TNF-*α* effect capable of crossing the intestinal barrier, and showed that LPS binding to Th1 cells and NF-*κ*B was significantly inhibited. Administration of lactic acid bacteria in prebiotic and probiotic forms can now be tailored to the needs of the patient. The influence on the immune system of individual bacterial strains has been conveniently compiled by Ljungh and Wadström [47]. An abbreviated listing is shown in Table 2. Lactic acid used in combination with *Saccharomyces boulardii*, a yeast found on lychee fruit, which has proven to be effective in preventing diarrhea and rdecreasing toxin A and toxin B, provides protection [48]. With this information, CAM practitioners may select lactic acid bacteria with *Saccharomyces boularddii* to enhance TGF-*β*-bearing regulatory cells and inhibit proinflammatory cytokine production. Therefore, *Lactobacillus*, other probiotic bacteria and their antigens may have a promise as new therapeutic agents for the treatment of many neuroimmune disorders that start in the GI tract and manifest in the nervous system.

### 4.5. Targeting NF-*κ*B

Part II of this series details the pathophysiology of intestinal epithelial tight junction permeability leading to the disruption of blood-brain barriers by Th17 cells, resulting in neuroinflammation. In this scenario, nuclear transcription factor kappa B (NF-*κ*B) is a powerful instigator of inflammation. Therefore, targeting NF-*κ*B may dampen neuroinflammation induced by Th17. Target genes of NF-*κ*B may be divided into different functional groups to reveal a predominance of those with primarily inflammatory functions. Kempe et al. [49] classified NF-*κ*B target genes and found that genes for monocyte, macrophage, neutrophils and T-cell chemoattractant chemokines and cytokines (RANTES, CCL20, CXCL3, CXCL2, CXCL11, CXCL6, MCP-1, IL-8 and GM-CSF) were up-regulated. Through the mitogen-activated protein kinase (MAPK) pathway, the phosphorylation and degradation of the NF-*κ*B-specific cytoplasmic inhibitor I*κ*B*α* and subsequent activation and nuclear translocation of NF-*κ*B is set in motion [50]. The initiation of NF-*κ*B activation occurs via cytokine signaling, innate or adaptive immune responses or environmental stressors. NF-*κ*B's key role is the regulation of immune responses to viral antigens or to bacterial antigens as is implicated in the opening of intestinal tight junctions [51, 52]. Activation of NF-*κ*B indicates down-stream interplay between the hypothalamic-pituitary-adrenal (HPA) axis and sympathetic nerve terminals as the endocrine response to psychological stressors and contributes to changes in the activity of the neuroendocrine axis to cellular behavior [49, 50].

Once the intersected activation pathways of NF-*κ*B are stimulated, an increase in certain proinflammatory cytokine levels is evident. This may involve TNF-*α*, IL-1*β* [51], IL-6 [53] and IL-8 [49, 52, 53]. Infections elicit the inflammatory response of IL-1*β*, which affects an increase in the expression of adhesion factors on endothelial cells to allow the transmigration of pathogen-fighting leukocytes to sites of infection. IL-1*β* is implicated in rheumatoid arthritis (RA) and enhanced intestinal permeability [51, 52]. Proinflammatory and anti-inflammatory IL-6 is detected in Castelman's disease, chronic lymphadenitis, Basedow's disease, Sjögren's syndrome and RA [54]. Secreted by any cells with toll-like receptors involved in the innate immune response, IL-8 functions to recruit neutrophils to phagocytose the antigen. High levels of IL-8 in pregnant women indicate a higher risk of inducing schizophrenia in offspring [55]. Each of these cytokines, IL-1*β*, IL-6, IL-8 and TNF-*α*, is capable of promoting the progression of inflammatory, autoimmune or neuroinflammatory disorders [51–55].

A multitude of complementary, and alternative, medicinal extracts affecting NF-*κ*B have been studied. Recent publications focusing on antioxidants to inhibit the activation of NF-*κ*B include aged garlic extract (allicin) [56], beta-carotene [57], curcumin [58] and quercetin [59]. Jiang et al. [60] concluded that lyceum seed oil is able to inhibit NF-*κ*B through the modulation of NF-*κ*B expression; while Rajakangas' group [61] studied nuclear translocation and found white currant to be an effective agent that inhibits NF-*κ*B. This transcription factor plays key roles in a variety of cellular processes ranging from inflammation to cancer, from differentiation to apoptosis and from immune response to proliferation. Acetyl-boswellic acids were used in a study [62] to show that in LPS-stimulated human peripheral monocytes, these molecules down-regulated TNF-*α* expression through the inhibition of NF-*κ*B. These finding suggest acetyl-boswellic acids as a tool for the development of novel therapeutic interventions. Therefore, the modulation of NF-*κ*B and associated cytokines such as IL-1*β*, IL6, IL-8 and TNF-*α* by using one or a combination of different herbal medicines may suppress Th17 and IL-17 production and inhibit neuroinflammation. CAM practitioners may thus consider NF-*κ*B modulation for therapeutic intervention or maintenance of neuroimmune inflammatory disorders.

### 4.6. Targeting TGF-*β*

A pleiotropic cytokine secreted by many cells, TGF-1*β*, plays vital neuroimmune roles. It can control cell growth, differentiation, inflammation, cell chemotaxis, apoptosis, hematopoiesis and plays a role in the protection of neurons from cell death induced by glutamate excitotoxic chemical hypoxia, apoptosis and oxidative injury [63]. Like its partner in the Th17 differentiation pathway, TGF-1*β* possesses bipolar characteristics. It is both a neuroprotective factor and a trigger of neuronal cell death [63]. This anti-inflammatory cytokine assists in T-cell differentiation. Under conditions of high concentration of TGF-*β*, which induces the expression of FoxP3, the naïve T-cell has been shown to differentiate into anti-inflammatory T_reg_, while in the presence of low levels, it will differentiate to proinflammatory Th17 [64–66]. Thus, by increasing the level of TGF-*β*, CAM practitioners may affect T-cell differentiation by polarizing the Th17/T_reg_ ratio toward anti-inflammatory T_reg_ for cases of inflammation and autoimmunity.

### 4.7. Targeting IL-6

Considered both pro- and anti-inflammatory, IL-6 plays a dual role. When secreted by T-cells and macrophages, IL-6 stimulates immune responses to trauma such as burns and tissue damage, and during bouts of exercise, muscle tissue cells will produce IL-6 [68]. Additionally, IL-6 can be produced by endothelial cells, retinal pigment epithelial cells, some tumor cells, astrocytes, fibroblasts, keratinocytes, bone marrow stromal cells and activated T and B cells [69–75]. In the presence of IL-6, low concentrations of TGF-*β* induce the expression of ROR*γ*t resulting in differentiation to Th17 and thus the secretion of volatile IL-17. Because of its inhibitory effects on TNF-*α* and IL-1 and activation of its interleukin-1 receptor agonist (IL-1RA) and IL-10, IL-6 can be defined as anti-inflammatory. Due to this duplicity, targeting IL-6 can be tricky. A balance of IL-6 is necessary to keep the level of Th17 cells down, while at the same time inhibiting TNF-*α* and IL-1. The use of bioflavonoids, previously described and summarized in Table 1, to block NF-*κ*B resulting in the down-regulation of IL-6 can bring down the cytokines involved in the inflammatory response [56–61].

### 4.8. Targeting Th17, ROR*γ*t Using Other Molecules

**Figure 1:**
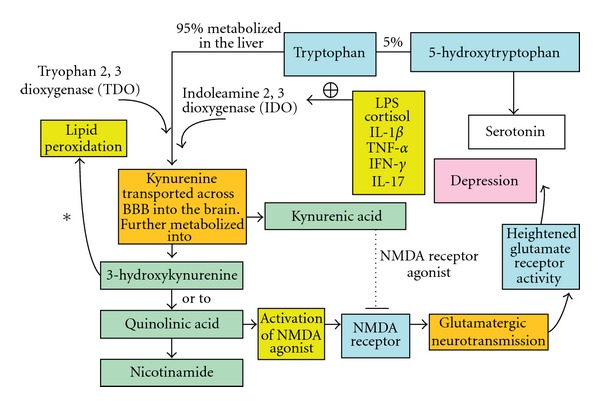
Degradation of tryptophan through the Kynurenine pathway by indolamine 2, 3 dioxygenase and its activation by highly inducible proinflammatory cytokines. Modified from Conklin et al. [67].

Innate agents that merit targeting include somatostatin and kynurenine with norepinephrine (NE). Naturally occurring in the body, somatostatin in gastrointestinal mucosa and kynurenines and NE in the nervous system, together these molecules play regulatory roles in inflammatory responses. The mucosal layer of the gastrointestinal tract is the body's first line of defense against environmental stressors. It is somatostatin's ability to reduce inflammation [76–78], which helps to keep the intestinal barrier functioning in its protective role. For this reason, CAM researchers may target somatostatin and kynurenine using tryptophan and hydroxytryptophan for down-regulation of neuroimmune disorders. Through meticulous studies [79, 80], researchers have elucidated the arduous pathways, both protective and toxic, of kynurenines and have found that by inhibiting the kynurenine pathway, brain damage from cerebral inflammation is reduced in animal models (see Figure 1). Natural molecules may be favored in some therapeutic interventions; however, targeting kynurenines, without upsetting the multiple neurotransmitter pathways they affect, can be very complicated.

### 4.9. Targeting Somatostatin

As regulatory hormone, somatostatin is expressed in the gastrointestinal tract (where nerve endings deliver somatostatin to the epithelial cells), pancreas and regions of the CNS. Classified as an inhibitory hormone, it has been shown to impede proinflammatory responses [81, 82]. Of particular interest, somatostatin secreted from non-neuronal cells along the digestive tract plays an important role as a mediator during mucosal inflammatory responses after physiological (induced by TNF-*α*) and pathophysiological (up-regulation of bacteria) stimulations [77]. Chowers et al. [77] found that somatostatin effectively inhibited spontaneously secreted IL-8 and IL-1*β*, as well as secretion initiated by TNF-*α* or bacterial invasion stimulation. More recently, Zavros et al. [78] concluded that circulating levels of somatostatin increased by infusion of IL-4, while IFN-*γ* suppressed the D-cell release of this peptide during inflammation; thus, Th1 predominant gastritis may be quelled through the up-regulation of somatostatin. Through reduced inflammation, the immunoregulatory function of somatostatin in the gastrointestinal tract may prevent the possibility of neurodegeneration that can occur after chronic intestinal barrier dysfunction.

Products of activated immune cells may exert their influence over the nervous system either by active transport of circulating cytokines into the CNS or via cytokine receptors located on the vagal nerve [83]. A key modulator of the sympathetic nervous system is the neurotransmitter norepinephrine (NE). Various levels of this neurotransmitter play an important role in anti- and proinflammatory biological responses. Increased NE, acting centrally, can contribute to the release of anti-inflammatory cytokines, whereas low concentrations support a proinflammatory biological state [84, 85]. Therefore, it has become crucial to determine neurotransmitter levels, as sub-optimal quantities favor potentially damaging proinflammatory activities. Considerable evidence has given credence to the important role of NE in manipulating adaptive immunity [86]. In a study involving mice, NE was required for the production of a Th1 cell-mediated immune response and perhaps further affected the development of Th1 [87]. Additionally, it has been proposed that NE is important in increasing the magnitude of a Th1 immune reaction [88]. Altogether, these studies indicate that NE may enhance the eventual production of Th1 cells [83]. Many studies have implicated IL-6 in contributing a key dose-dependent role in sympathetic nerve activity. Specifically, the combined concentrations of sub-threshold IL-6 and IL-1*β* can attenuate sympathetic nerve activity [83]. Under stress-induced situations, where optimal NE levels were present, NE attenuated the release of TNF-*α* along with IL-6 [86]. Taken together not only can IL-6 and TNF-*α* augment, attenuate or silence in their effects on rate of release of NE [89], but it is decisively clear that NE itself can significantly influence whether a system is more prone to anti- or proinflammatory responses.

**Figure 2:**
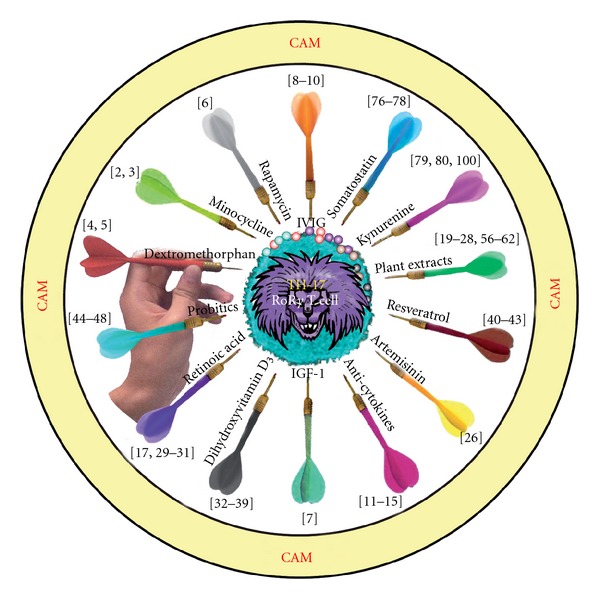
Various CAM intervention methods with appropriate references for targeting TH17 ROR*γ*t cells in autoimmune disorders or neurodegenerative diseases are shown. Possibilities range from pharmaceuticals to natural plant extracts and innate molecules.

NE is responsible for most of the activity within the sympathetic nervous system. It mediates the inhibition of T- and B-cell activations, inhibits IL-2-generated lymphocyte activation and modulates keratinocyte mitogenesis [86, 90, 91]. Low levels of NE promote a proinflammatory state, while the up-regulation of NE favors anti-inflammatory cytokines [92]. Interestingly, all kynurenines and resultant intermediates can be measured in blood and urine [79]; therefore, targeting kynurenines in conjunction with NE measurements will provide an invaluable therapeutic advantage for healthcare practitioners to achieve the best patient results. In patients with neuroinflammation and autoimmunity, these intervention methods for targeting Th17 ROR*γ*t cells ranging from pharmaceutical to plant extracts and other molecules with appropriate references are shown in Figure 2.

## 5. Conclusion

During the past 30 years, discussions about the limitations of Th1/Th2 implications for health and disease have resulted in several major revisions of the hypothesis, and hence, the identification of T_reg_ and Th17 cells. Currently, these subsets of CD4^+^ T-helper cells are classified based upon unique cytokine products, signaling pathways and lineage-specific transcription factors. However, recent data argue for more complexity and flexibility in these subsets than was previously assumed [93]. As with any model, flexibility and plasticity of the T-cell subset are the subject of intense investigation and enhancement. As was concluded by Wilson et al. [94], “a future challenge will be to determine with greater clarity how specific combinations of epigenetic modifications are established by networks of lineage-specifying transcription factors, and whether, when and how they can later be removed or selectively modified to achieve or alter T_H_-lineage specification”.

Thus, based on the best available evidence, the modification of T_H_-lineage requires a full understanding of the neuroimmune inflammatory pathway, which is necessary to combat inflammation, autoimmunity and neurodegeneration. In this regard it is vital to understand the significant roles played in neuroinflammation by Th17 and its effector cytokine, IL-17, in conjunction with IL-23, IL-27 and Th1 autoreactive cells.

Although IL-17 is highly pathogenic, as evidenced by its presence in a variety of inflammatory disorders, its initial intent is not destructive. Th17 plays a protective role in host defense against infection, by inducing chemokine and G-CSF expression from surrounding cells thereby recruiting neutrophils and macrophages to infected tissues [16, 95]. Within the intestinal mucosa, naturally occurring Th17 cells control a variety of bacterial and fungal infections at the mucosal surfaces [95–98]. It is in the presence of chronic inflammation that Th17 becomes destructive and over produces IL-17 [98, 99].

Through careful manipulation of the ROR*γ*t differentiation of naïve T cells into the Th17 cells cascade using the different CAM modalities described in this manuscript, a balanced immune response can be achieved. Research and technology have defined diverse therapeutic options, which have been mentioned above. With a variety of choices available, therapy can be tailored to fit the individual patient's needs. Whether using pharmaceutical or natural treatments, it is important to establish the patient's level of tissue-specific antibodies, status of immune function, including cytokine production before commencing therapy. Such laboratory assessments should be repeated periodically to monitor the effectiveness of the treatment protocol. Alterations may be required to recover from the inflammation inherent in autoimmune, neuroimmune and neurodegenerative diseases and return to a state of optimal health. More research and publications are needed before a proper meta-analysis of compiled data can be professionally assessed. However, the small number of studies presented in this manuscript shows encouraging experimental evidence, which may inspire the execution of larger, longitudinal research projects in the near future. This additional information could yield detailed cross-comparisons of integrative and CAM therapies for inflammatory and autoimmune disorders.

## Figures and Tables

**Table 1:** Natural alternatives for reducing inflammation in autoimmune conditions.

Agent	Reference	Result
Moutan cortex	[19]	Reduced concentration of TNF-*α*, IL-1*β* and IL-6 in LPS-stimulated RAW264.7 cells
Moutan cortex	[20]	Increased production of IL-10 and a decrease in level of proinflammatory cytokines
*Perillae Fructus*	[21]	Suppressed IL-4 production, inhibiting IgE secretion; reduced IL-5 and IL-13; suppressed excess Th2 activity and rebalanced Th1/Th2
Atractylenolide I	[22]	Up-regulate TNF; down-regulate IL-1
Tibetan medicated-bath	[23]	Decreased IL-6 and TNF-*α* levels
*Opuntia humifusa* Raf	[24]	Blocked expression of IL-6 from LPS-stimulated RAW264.7 cells
*Total Flavonoids of Fructus Chorspondiatis*	[25]	Inhibited NF-*κ*B signaling pathway
*Artemisia annua* L.	[26]	Inhibited IL-17A production; reduced mRNA expression of ROR*γ*t; reduced IL-6 production
Kummerowia striata (Thunb.) Schindl ethanol extract	[27]	Suppressed NF-*κ*B activation; up-regulated IL-10 and HO-1
*Artemisia alba*	[28]	Inhibited IL-1-induced NF-*κ*B and AP-1 activation
Ginger extract from *Alpinia gallanga* and *Zingiber officinale*	[28]	Reduced level of mRNA
*Longicera japonica*	[28]	Inhibited LPS-induced degradation of I-*κ*B*α* and induction of NF-*κ*B p65, TNF-*α* and inducible NO synthase (iNOS)
*Phlebodium decumanum*	[28]	Reduced TNF production; inhibited IL-6 production; increased release of TNF-R2 and IL-1 receptor agonist
*Scandix austalis* extracts	[28]	Inhibited IL-1-induced NF-*κ*B and AP-1 activation
*Tripterygium wilfordii*	[28]	Lowered the inflammatory response
*Urtica dioica* leaf extracts	[28]	Inhibited NF-*κ*B

**Table 2:** Some individual bacterial strains that influence the immune system [47].

Strain	Effect
*L. casei* Shirota	Induced IL-12 production
Heat-killed *L. casei* and *L. fermentum*	Induced TNF-*α* production
*L. paracasei* Ncc2461	Induced CD4^+^ T-cells to produce IL-10 and TGF-*β*
*L. rhamnosus* GG with LPS	Inhibited TNF-*α*, but not IL-10
*L. reuteri* + *L. brevis*	Induced TNF-*α*, IL-2, IL-1*β*
*L. rhamnasus* + *L. acidophilus* + *B. lactic*	Enhanced immunoreactivity of spleen cells and phagocytes
*L. acidophilis* + *L. delbrueckii* ssp *bulgaricus* + *B. bifidum*	Produced an anti-inflammatory response
*L. casei* Shirota	Inhibited translocation of NF-*κ*B and production of IL-6

L., Lactobacillus; B., Bifidobacterium.
